# Are European Soccer Players Worth More If They Are Born Early in the Year? Relative Age Effect on Player Market Value

**DOI:** 10.3390/ijerph17093301

**Published:** 2020-05-09

**Authors:** Benito Perez-Gonzalez, Alvaro Fernandez-Luna, Daniel Castillo, Pablo Burillo

**Affiliations:** 1 Faculty of Health Sciences, Universidad Isabel I, Calle de Fernán González, 76, 09003 Burgos, Spain; benito.perez@ui1.es (B.P.-G.); daniel.castillo@ui1.es (D.C.); 2 Faculty of Sports Sciences, Universidad Europea de Madrid, Calle Tajo S/N, 28670 Madrid, Spain; pablo.burillo@universidadeuropea.es

**Keywords:** economic value, relative age effect, soccer

## Abstract

The relative age effect (RAE) consists of the lower presence of members of an age group born in the months furthest from the age cut-off date established. In youth soccer, it is known that because of this effect the birth dates of more players in a team are closer to the cutoff of 1 January. These older players, due to their physical and psychological advantages, are more likely to be identified as talent. This study aimed to examine whether RAE can be identified in professional players of the top five European soccer leagues (Spain, Italy, England, Germany, and France) and to assess its influence on the perceived market value of the players. Market value data for 2577 players were obtained from the Transfermarkt database. A significant RAE was produced in all leagues (*p* < 0.05). However, this bias did not affect the market value of the professional elite soccer players examined here. Our observations indicate that, while the identification and promotion of talent at young ages are often biased by RAE, once players have reached the professional stage, the market value assigned to them is based more on factors other than their date of birth.

## 1. Introduction

The relative age effect (RAE) consists of a decline in the frequency of members born in the last months leading up to the cutoff after which a new age category is established. While RAE occurs in different settings, in the sport or school setting, this effect will mean that in a given age group, there will be more older athletes or children with advantages in athletic or academic endeavors than younger ones. Early studies examining this effect in sport were those of Barnsley, Thompson, and Barnsley [1], who noted that 40% of juvenile category ice hockey players in Canada had been born in the first quarter of the year. In soccer, the first study addressing this issue was that by Barnsley, Thompson, and Legault [2]. In 1994, Dudink [3] confirmed that RAE existed in the Dutch and English youth soccer league. Subsequently, Musch and Grondin [4] analyzed the effect in 57 studies focusing on 11 different sports. Their conclusion was that when there are large numbers of athletes, selection based on technical capacity and not on criteria of physical development is difficult. Cobley, Baker, Wattie, and McKenna [5] determined the characteristics of an athlete most affected by RAE were being male, aged 15–18 years, and involved in competition sports with a high critical mass of practitioners. Between the ages of 15 to 18, individuals may have different levels of maturity affecting their competitive ability. In elite groups of these juvenile categories, there is usually an overrepresentation of people with a high level of physical maturity [6]. In this respect, Katsumata, Omuro, Mitsukawa, and Nakata [7] found that biological maturity occurs earlier in elite elementary and junior high school players than in recreational elementary and junior high school players. An interesting proposal to minimize RAE and other problems related to the physical development of athletes in popular sports is bio-banding. This approach consists of grouping youth athletes into ‘bands’ or groups based on characteristics other than chronological age [8]. Cumming et al. [9] and Bradley et al. [10] report some benefits of grouping youth athletes according to physical maturity.

According to the literature, it has been shown that RAE similarly affects the top European leagues: LaLiga (Spain), Premier League (England), Serie A (Italy), Bundesliga (Germany) and Ligue 1 (France). All feature a greater proportion of members born in the first quarter of the year. The presence in the 2009–2010 season of RAE in the Italian, Spanish, and French leagues was confirmed both in a study [11], and cohort of 841 professional players competing in international Union of European Football Associations tournaments (UEFA) [12]. Yagüe et al. [13] extended the study of RAE to the ten most important European soccer leagues in the season 2016–2017 (*N* = 5201) confirming a RAE in all of them except the Belgian league. These authors also confirmed RAE in all field positions, being especially high in midfielders and reduced, though still present, in forwards. In the Italian league, the effect continues to exist [14]. The authors Saavedra-García, Matabuena, Montero-Seoane, and Fernández-Romero also noted a RAE over more than a century (1908–2012) in a study of FIFA male competitions [15]. In Spanish soccer, over the period 2000 to 2011, the presence of RAE was confirmed both in top and lower categories and also for defender and midfield positions [16]. In a subsequent Spanish study, findings were similar and it was found that 60% of players were born in the first semester of the year. Furthermore, in club youth teams, the figure of 75% born in the first six months of the year has recently increased to 80% [17].

The age effect not only influences first division soccer teams, it also has an impact on lower level players who compete in the second divisions of the five top European teams [18] and the US college soccer tournament of the National Collegiate Athletic Association (NCAA) [19]. In this last study of the 2014–2015 NCAA season, it was found that that RAE prevented second opportunities even for lower level players, such as those in second division teams. Some studies [20] have suggested that players that initially experience maturity disadvantages in major sports may have a second opportunity to do well in professional minor sports (i.e., futsal vs. soccer). This explains a reverse RAE in futsal players, and could also explain this contrary effect described in forwards in Australian rugby union players [21]. Further, a new line of research has detected RAE at the end of the professional stage in Italian senior soccer [15]. It also seems that RAE mainly manifests in sports of a large critical mass of athletes who aspire to be selected and promoted to a higher level. This explains why it has been difficult to identify RAE in minority sports, as described by Lupo et al. [22]. However, some studies have also revealed RAE in less popular sports [23,24]. A cumulative effect of RAE over the years in the different countries is common yet. Nevertheless, some studies suggest that players born in the last months of the year could even attain higher professional levels than relatively older players [25,26].

In the sports competition market, teams strive to acquire the best talents available for their income possibilities. In terms of demand theory [27], soccer players may be viewed as goods that access a market of limited demand which would correspond to established professional category teams. Ashworth and Heyndels [28] found that German professional soccer players born late after the cut-off date systematically had higher wages than their older fellow players, but not in all positions. From an economics perspective, RAE reduces promotion opportunities by following incorrect criteria, which reduces the optimal average value of the players promoted [6].

Doyle and Bottomley [29], analyzed two sets of data for elite soccer players (the top 1000 players in the 2013–2014 season and UEFA Youth League Under 19 participants of the 2014–2015 season) using data related to birth date and market value as the first study to use economic assessment data from the database “Transfermarkt”. In both sets, RAE could be identified in terms of frequency but not market value. In other words, while there are more players who are born early in the year their transfer value is not higher and neither are they conferred more game time.

From 2013–2014 to the present, there has been a high increase in club incomes, player transfers, continental competition awards, and also a commercial expansion worldwide of the main European clubs [30]. This much higher income has been translated to the market values of players which have risen in this four year period at a much higher rate than inflation in the Euro zone countries. The mean variation produced in the value of the players in the 10 clubs that featured the higher values in Transfermarkt over this period was 91.8%, i.e., players have almost doubled their market value in only four seasons [31] (Table 1).

The larger amounts of money available to clubs is mainly used for two purposes: paying player salaries and acquiring new players (transfers). Frick [32] argued that a player′s salary was influenced by his age and experience and was more related with his past activity than his potential future performance. Along these lines, other studies have shown that market value is related to performance indicators such as pass accuracy and numbers of assists, tackles, or goals [33], that is, the players’ performance in lower categories.

While several studies have explored the effects of different variables on the perceived market value of soccer players, this topic is still far from being exhausted. Specifically, few studies in the area of perceived market value have addressed the impacts of the phenomenon RAE [29], identified in numerous sports. Thus, given the high and increasing costs of player transfers in European soccer and of identifying young talent, this study sought to examine whether a RAE is present in the five top European soccer leagues (Spain, England, Italy, Germany, and France) and to assess its possible influence on the market value of players.

## 2. Materials and Methods

Participants were professional soccer players with FIFA Pro Player cards included in the Transfermarkt database (*N* = 2577). Lower category players within a team were excluded if they had played for less than 90 min in professional category matches. The average age of the players was 30 ± 4.3 years.

The Transfermarkt website was used as the source for market values (in € million) of the 2577 players comprising the Spanish (LaLiga), French (Ligue1), German (Bundesliga), Italian (Serie A), and English (Premier League) soccer leagues. This source has also been used in recent studies [34] as providing reliable game performance indicators and has been described as a good predictor of real market values according to Herm et al. [33].

We conducted an exhaustive search of the market values of players in first division soccer leagues. To avoid bias, this was done at mid-season, once summer and winter markets for the 2017–2018 season had closed. The exact date of data collection was 31 January 2018. The official web pages of the clubs were used to obtain players’ dates of birth.

The RAE was detected through Poisson regression [29,35]. The Poisson regression formula y = e (b_0_ + b_1x_) serves to explain the frequency count of an event (y) by an explanatory variable x. The data used for Poisson regression were week of birth (W_B_; whereby the first week in January was designated W_B 1_), and time period of birth (t_B_; describing how far from the beginning of the year a player was born0. This last index ranging between 0 and 1 was calculated as t_B_ = (W_B_ − 0.5)/52. In the Poisson regression, the event (y) was the frequency of birth in a given week and the explanatory variable (x) was t_B_. We also calculated the index of discrimination (ID) according to Doyle and Bottomley as e-b_1_ [35]. This index measures the relative odds of a player born on day 1 versus day 365 of the competition year being selected. The likelihood ratio R^2^ was determined according to Cohen et al. [36].

Once we had assessed the presence or absence of RAE, we performed the Kolmogorov–Smirnov test to determine if the economic value of players followed a given distribution (*p* < 0.01). Then we compared the market value of players by quarter of birth using a non-parametric Kruskal–Wallis test with a Bonferroni–Dunn post-hoc test, and by semester of birth using the non-parametric Mann–Whitney U statistic. In addition, to assess whether the time of birth has an effect on market value, we conducted an odds ratio analysis of the impacts of birth quarter or semester on perceived player market value. The perceived market value is described as medians 1 and 2 (< or >3 € million) or tertiles 1 and 3 (0.1–1.5 € million and >6.1 € million). These medians and tertiles were obtained from all the market values of the sample. All statistical tests were performed using the software package SPSS version 20 (Statistical Package for Social Science) for Windows (SPSS Inc., Chicago, IL, USA). Significance was set at *p* < 0.05.

## 3. Results

Birth date distributions by quartile (Q) and semester (Se) for the players in the different leagues are provided in Table 2. Our Poisson regression by frequency analysis revealed the presence of a significant overall RAE across all leagues. The effect was not significant for goalkeepers in all leagues, but for other positions we detected a RAE in some leagues including defenders participating in LaLiga, Bundesliga, and Serie A; midfielders in LaLiga and Serie A; and forwards in Bundesliga (Table 3).

Table 4 provides descriptive data of the estimated average value (€ million) of European League soccer players according to their quarter (Q) or semester (Se) of birth. We found no differences in market value according to the quarter or semester of birth in all the leagues analyzed (*p* < 0.05). Table 5 shows player market values as medians (median 1 = 0.1–3 M€; median 2 ≥ 3.1 M€) and tertiles (tertile 1 = 0.1–1.5 M€; tertile 3 ≥ 6.1 M€) by player semester of birth. In most cases, it may be observed that the higher percentages of players appear in median 1 or tertile 1 across all leagues, with the exception of the Premier League, where most players occur in the median and tertile with the highest market values, and LaLiga, where there is a higher percentage of players in the third tertile. However, no significant differences emerged in any case in terms of perceived value depending on the semester of birth. Notwithstanding, our risk analysis (odds ratios) revealed no significant relationship between semester of birth and being included in the medians and tertiles of the highest perceived values.

## 4. Discussion

Our frequency analysis revealed the presence of a significant overall RAE in the top five European soccer leagues. Recruitment age is critical as it will substantially affect the future of those selected or not at that moment. Our findings confirm that professional soccer is affected by the core selection which does not show an equal distribution of those born in the different months of the year as would be the case according to the law of large numbers in a large randomly distributed sample.

In their study, Rada et al. [18] compiled solutions to the RAE proposed by the different authors. The first of these, based on the findings of Gerdin, Hedberg, and Hageskog, is that competitions should have no classifications until beyond the juvenile stage [37]. Another proposal emerging from a study by Lagestad, Steen, and Dalen [38] is that it should be ensured that at least 40% of youth team players are those born in the second semester of the year. The idea proposed by Barnsley and Thomson [39] was to reduce the maximum relative age range in the different categories, for example by organizing competitions by semester rather than calendar year of birth.

According to Hill and Sotiriadou [40], RAE can be mitigated in two ways. The first consists of the education of trainers, teachers, and generally anyone who needs to make decisions about the sports performance of children and adolescents, so that attempts are made to reduce the effect through more flexibility and observation. The second way is rotation of the cutoff date over the years so that the same subjects will not always be affected. This second proposal is difficult to apply as our education system and sport competitions are mostly organized by calendar year. Webdale, Baker, Schorer, and Wattie [41] compared 63 peer-reviewed reports of RAE solutions. These authors concluded that most proposals to reduce RAE are theoretical and there has been no attempt to implement them. We recommend that when drawing up teams, coaches should consider birth month as another variable to be taken into account. In this context, bio-banding could be interesting for youth soccer academies [8,9]. Several applications of bio-banding in youth soccer have generated positive responses of players and coaches [7]. As mentioned above, the incidence of RAE is greater in sports with a sufficiently high critical mass. Nevertheless, some studies have found no RAE impacts in major sports such as NFL (American National Football League) or NBA [42].

In terms or market value of players, our observations confirm the results of the study by Doyle and Bottomley [29] conducted on data for the season 2013–2014 in the top 1000 Transfermarkt value players at that time. These authors noted that players born early in the year had greater opportunities due to the assessment bias whose effects are multiplied by the different agents in the selection process [43]. However, market values tend to naturally balance out. In other words, once they have achieved senior professional soccer categories, the value of players based on their sports performance naturally evens out considering this performance as a main attribute for market value. Therefore, market value at this stage is already independent of chronological age (for example, 24, 25, or 26 years) or time of birth (month or semester). However, given the existence RAE, it is likely that there will be inefficiency in the selection of talent. Accordingly, we could consider that the offer of players of given characteristics does not reach its optimum level (efficient selection), which in soccer, could induce an inflation effect due to an excess of demand. In contrast, by eliminating RAE, the mean quality of maximum category players would improve, and this would improve the competitive balance and reduce the transfer market′s tension. To gain further insight into this issue, studies are needed to establish the economic value of players before their professional stage and to follow these players in a longitudinal study. It would also be interesting to determine whether the starting date of a professional player may be conditioned by RAE.

As the main limitation of our study, there are several variables not included in our study that could affect perceived market value such as a player′s popularity [33] and social-media activity [44]. Another factor that could generate bias is the influence of income from broadcasting rights in different European Leagues, which will directly affect the transfer value of players [45] and indirectly their perceived market value. Thus, if all leagues had equal income from broadcasting, this would especially affect non-top teams and limit differences in market value caused by RAE. Finally, we should highlight, as have many other authors [46,47], the need for a full estimate model of the real market value of players in their different stages of development. Only then will we observe a clear relationship between RAE and market value.

## 5. Conclusions

This study sought to examine the prevalence of RAE in European soccer and to assess its impacts on the market value of players. To this end, we examined the birth dates and market values of professional soccer players in the five top leagues. A RAE was noted in all these leagues, especially in defense players. Our findings also indicate no significant differences in market value according to a player′s date of birth.

According to the data collected in this large population sample of professional soccer players (*N* = 2577), it would seem that despite a RAE bias among players during their stages of growth and development, once players have been selected for maximum competition levels, it is their talent that conditions their market value at any given time. Thus, we could say that the market value assigned to a professional soccer player is democratic, while this is not the case for their identification and promotion during their journey towards elite categories.

## Figures and Tables

**Table 1:** Trends in player mean market values over the period 2013–2018.

Team	Competition	Value/Player M€ 2013–2014	Value/Player M€ 2017–2018	% Variation
FC Barcelona	LaLiga	25.4	48.6	91.3%
Real Madrid CF	LaLiga	23.6	44.4	88.0%
Manchester City	Premier League	20.8	41.4	99.0%
FC Paris Saint-Germain	Ligue 1	17.8	36.1	102.2%
Tottenham Hotspur	Premier League	12.4	30.0	142.2%
Atlético de Madrid	LaLiga	10.7	33.7	215.4%
Chelsea FC	Premier League	20.0	33.6	68.0%
Manchester United	Premier League	19.7	30.7	55.6%
FC Bayern Munich	Bundesliga	22.0	35.4	60.5%
Juventus	Serie A	14.9	25.7	72.2%
	Mean	18.7	35.9	91.8%

Data source: Transfermarkt (2018) for player market values in 2013/14 and 2017/18. All data are referred to constant prices of June 2018 according to the consumer price index. Million Euros (M€); FC and CF mean football club.

**Table 2:** Birth date distributions of European league soccer players (2017/2018) according to their quartile (Q) or semester (Se) of birth.

		Q1	Q2	Q3	Q4	Se 1	Se 2
LaLiga	*n*	151	128	109	95	279	204
	%	31.3%	26.5%	22.6%	19.7%	57.8%	42.2%
Premier league	*n*	144	132	120	113	276	233
	%	28.3%	25.9%	23.6%	22.2%	54.2%	45.8%
Bundesliga	*n*	146	134	131	81	280	212
	%	29.7%	27.2%	26.6%	16.5%	56.9%	43.1%
Serie A	*n*	194	137	112	85	331	197
	%	36.7%	25.9%	21.2%	16.1%	62.7%	37.3%
Ligue 1	*n*	187	127	128	101	302	241
	%	34.4%	23.4%	23.6%	18.6%	55.6%	44.4%
All players	*n*	822	658	600	475	1468	1087
	%	32.2%	25.8%	23.5%	18.6%	57.5%	42.5%

**Table 3:** Poisson regression analysis of relative age effect (RAE) by frequency for all players and the different field positions.

		LaLiga (*n* = 477)	Premier League (*n* = 511)	Ligue 1 (*n* = 537)	Bundesliga (*n* = 500)	Serie A (*n* = 529)
**Overall**	**W_B_**	24 ± 15	25 ± 15	23 ± 15	24 ± 14	22 ± 15
	**t_B_**	0.45 ± 0.29	0.47 ± 0.30	0.44 ± 0.29	0.46 ± 0.28	0.42 ± 0.28
	**b_0_**	2.524	2.446	2.680	2.517	2.766
	**b_1_**	−0.606	−0.331	−0.735	−0.530	−0.969
	**ID**	1.83	1.39	2.09	1.70	2.64
	**R^2^**	0.49	0.93	0.69	0.84	0.55
	***p*-value**	<0.001	0.031	<0.001	<0.001	<0.001
**Goalkeeper**	**W_B_**	23 ± 15	23 ± 15	23 ± 15	26 ± 14	19 ± 14
	**t_B_**	0.44 ± 0.30	0.43 ± 0.29	0.44 ± 0.29	0.49 ± 0.27	0.35 ± 0.27
	**b_0_**	0.449	0.826	0.691	0.603	0.983
	**b_1_**	−0.168	−0.573	−0.429	−0.70	−0.918
	**ID**	1.18	1.77	1.53	1.07	2.50
	**R^2^**	0.99	0.93	0.96	0.99	0.86
	***p*-value**	0.733	0.205	0.320	0.881	0.061
**Defender**	**W_B_**	24 ± 16	24 ± 15	25 ± 15	23 ± 14	23 ± 14
	**t_B_**	0.45 ± 0.30	0.46 ± 0.29	0.47 ± 0.29	0.42 ± 0.27	0.43 ± 0.28
	**b_0_**	1.522	1.509	1.464	1.452	1.721
	**b_1_**	−0.632	−0.513	−0.474	−0.584	−0.954
	**ID**	1.88	1.67	1.61	1.79	2.60
	**R^2^**	0.89	0.92	0.032	0.91	0.76
	***p*-value**	0.018	0.046	0.94	0.045	<0.001
**Midfielder**	**W_B_**	24 ± 14	25 ± 16	23 ± 15	24 ± 15	22 ± 15
	**t_B_**	0.45 ± 0.27	0.47 ± 0.31	0.43 ± 0.29	0.45 ± 0.28	0.42 ± 0.28
	**b_0_**	1.212	1.193	1.470	1.131	1.547
	**b_1_**	−0.585	−0.423	−0.535	0.006	−0.727
	**ID**	1.80	1.53	1.71	0.99	2.07
	**R^2^**	0.92	0.95	0.037	0.99	0.87
	***p*-value**	0.06	0.156	0.92	0.984	0.011
**Forward**	**W_B_**	24 ± 15	27 ± 15	22 ± 14	25 ± 14	24 ± 15
	**t_B_**	0.46 ± 0.29	0.51 ± 0.29	0.41 ± 0.28	0.48 ± 0.27	0.45 ± 0.28
	**b_0_**	1.392	0.821	1.455	1.196	1.218
	**b_1_**	−0.52	0.433	−0.909	−0.353	−0.471
	**ID**	1.68	0.65	2.48	1.42	1.60
	**R^2^**	0.92	0.95	0.83	0.97	0.95
	***p*-value**	0.076	0.152	<0.001	0.228	0.145

W_B_: week of birth; t_B_: time of birth; ID: index of discrimination.

**Table 4:** Estimated average value (€ million) of European League soccer players (2017/2018) according to their quarter (Q) or semester (Se) of birth.

		Q1	Q2	Q3	Q4	*p*-Value	Se 1	Se 2	*p*-Value
**LaLiga**	***n***	151	128	109	95		279	204	
	**Value**	10.77	10.08	6.79	7.71	0.21	10.45	7.22	0.59
	**SD**	18.95	20.76	12.50	12.11		19.77	12.30	
**Premier league**	***n***	144	132	120	113		273	233	
	**Value**	11.31	14.05	12.06	12.57	0.55	12.62	12.30	0.60
	**SD**	13.85	19.83	14.94	15.49		17.00	15.18	
**Bundesliga**	***n***	146	134	131	81		280	212	
	**Value**	6.10	5.77	6.90	6.00	0.76	5.94	6.58	0.30
	**SD**	9.61	7.79	11.32	7.54		8.77	10.04	
**Serie A**	***n***	194	137	111	85		331	197	
	**Value**	7.12	6.49	5.20	7.69	0.33	6.86	6.27	0.92
	**SD**	10.44	10.70	7.11	13.50		10.54	10.48	
**Ligue 1**	***n***	187	127	128	101		302	241	
	**Value**	6.17	2.98	4.85	5.80	0.12	5.00	5.09	0.33
	**SD**	15.39	4.18	7.71	15.39		12.48	11.45	
**All players**	***n***	824	658	600	475		1468	1075	
	**Value**	8.11	7.88	7.17	8.16	0.55	8.07	7.54	0.82
	**SD**	14.11	14.68	11.36	13.58		14.42	12.34	

Significance set at *p* < 0.05.

**Table 5:** Median (median 1 = 0.1–3 M€; median 2 ≥ 3.1 M€) and tertile (tertile 1 = 0.1–1.5 M€; tertile 3 ≥ 6.1 M€) market values and odds ratio analysis by player semester of birth (Se 1, Se 2).

		Median 1	Median 2	*p*-Value	Odds Ratio	Tertile 1	Tertile 3	*p*-Value	Odds Ratio
**LaLiga**	**Se 1 (*n*)**	142	137	0.07	0.711 (0.49–1.02)	86	94	0.08	0.66 (0.49–1.04)
	**%**	50.9	49.1			47.8	52.2		
	**Se 2 (*n*)**	121	83			77	56		
	**%**	59.3	40.7			57.9	42.1		
**Premier League**	**Se 1 (*n*)**	81	195	0.48	1.17 (0.79–1.53)	49	140	0.53	0.96 (0.60–1.52)
	**%**	29.3	70.7			25.9	74.1		
	**Se 2 (*n*)**	61	172			38	130		
	**%**	26.2	73.8			22.6	77.4		
**Bundesliga**	**Se 1 (*n*)**	149	131	0.71	1.07 (0.75–1.53)	96	73	0.64	1.14 (0.72–1.81)
	**%**	53.2	46.8			56.8	43.2		
	**Se 2 (*n*)**	109	103			70	61		
	**%**	51.4	48.6			53.4	46.6		
**Serie A**	**Se 1 (*n*)**	189	142	0.92	0.96 (0.67–1.38)	126	102	0.57	0.86 (0.55–1.33)
	**%**	57.1	42.9			55.3	44.7		
	**Se 2 (*n*)**	114	83			76	53		
	**%**	57.9	42.1			58.9	41.1		
**Ligue 1**	**Se 1 (*n*)**	198	104	0.71	1.07 (0.79–1.53)	142	58	0.53	1.197 (0.73–1.95)
	**%**	65.6	34.4			71	29		
	**Se 2 (*n*)**	154	87			112	44		
	**%**	63.9	36.1			72.8	28.2		
**All Leagues**	**Se 1 (*n*)**	749	709	0.9	1.01 (0.86–1.18)	499	467	0.88	0.98 (0.81–1.19)
	**%**	51.7	48.3			51.7	48.3		
	**Se 2 (*n*)**	559	528			373	344		
	**%**	51.4	48.6			52	48		

Significance set at *p* < 0.05.

## References

[1] Barnsley R.H., Thompson A.H., Barnsley P.E. (1985). Hockey success and birthdate: The RAE. Canadian Association for Health. Phys. Educ. Recreat..

[2] Barnsley R.H., Thompson A.H., Legault P. (1992). Family Planning: Football Style. The Relative Age Effect in Football. Int. Rev. Sociol. Sport.

[3] Dudink A. (1994). Birth date and sporting success. Nature.

[4] Musch J., Grondin S. (2001). Unequal Competition as an Impediment to Personal Development: A Review of the Relative Age Effect in Sport. Dev. Rev..

[5] Cobley S., Baker J., Wattie N., McKenna J. (2009). Annual age-grouping and athlete development: A meta-analytical review of relative age effects in sport. Sports Med..

[6] Castillo D., Pérez-González B., Raya-González J., Fernández-Luna A., Burillo P., Lago-Rodríguez A. (2019). Selection and promotion processes are not associated by the relative age effect in an elite Spanish soccer academy. PLoS ONE.

[7] Katsumata Y., Omuro K., Mitsukawa N., Nakata H. (2018). Characteristics of Relative Age Effects and Anthropometric Data in Japanese Recreational and Elite Male Junior Baseball Players. Sports Med. Open.

[8] Malina R.M., Cumming S.P., Rogol A.D., Coelho-e-Silva M.J., Figueiredo A.J., Konarski J.M., Koziel S.M. (2019). Bio-Banding in Youth Sports: Background, Concept, and Application. Sports Med..

[9] Cumming S.P., Brown D.J., Mitchell S., Bunce J., Hunt D., Hedges C., Crane G., Gross A., Scott S., Franklin E. (2018). Premier League academy soccer players’ experiences of competing in a tournament bio-banded for biological maturation. J. Sports Sci..

[10] Bradley B., Johnson D., Hill M., McGee D., Kana-ah A., Sharpin C., Shapr P., Kelly A., Cumming S.P., Malina R.M. (2019). Bio-banding in academy football: Player’s perceptions of a maturity matched tournament. Ann. Hum. Biol..

[11] Salinero J.J., Pérez-González B., Burillo P., Lesma M.L. (2013). El efecto de la edad relativa en el fútbol español. Apunt. Educ. Física Deportes.

[12] González-Víllora S., Pastor-Vicedo J.C., Cordente D. (2015). Relative Age Effect in UEFA Championship Soccer Players. J. Hum. Kinet..

[13] Yagüe J.M., de la Rubia A., Sánchez Molina J., Maroto-Izquierdo S., Molinero O. (2018). The Relative Age Effect in the 10 Best Leagues of Male Professional Football of the Union of European Football Associations (UEFA). J. Sports Sci. Med..

[14] Brustio P.R., Lupo C., Ungureanu A.N., Frati R., Rainoldi A., Boccia G. (2018). The relative age effect is larger in Italian soccer top-level youth categories and smaller in Serie A. PLoS ONE.

[15] Saavedra-García M., Matabuena M., Montero-Seoane A., Fernández-Romero J.J. (2019). A new approach to study the relative age effect with the use of additive logistic regression models: A case of study of FIFA football tournaments (1908–2012). PLoS ONE.

[16] Salinero J.J., Pérez-González B., Burillo P., Lesma M.L. (2013). Relative age effect in European professional football. Analysis by position. J. Hum. Sport Exerc..

[17] Salinero J.J., Pérez-González B., Burillo P., Lesma M.L., Herrero H. (2014). El efecto de la edad relativa en el fútbol profesional español/Relative age effect in Spanish professional football. Rev. Int. Med. Cienc. Act. Física Deporte.

[18] Rađa A., Padulo J., Jelaska I., Ardigò L.P., Fumarco L. (2018). Relative age effect and second-tiers: No second chance for later-born players. PLoS ONE.

[19] Hurley E., Comstock B.A., Haile L., Beyer K.S. (2019). Relative age effect in collegiate soccer: Influence of nationality, playing position, and class. J. Strength Cond. Res..

[20] Lago-Fuentes C., Rey E., Padrón-Cabo A., Prieto-Troncoso J., Garcia-Núñez J. (2020). The Relative Age Effect in Professional Futsal Players. J. Hum. Kinet..

[21] Jones B.D., Lawrence G.P., Hardy L. (2018). New evidence of relative age effects in “super-elite” sportsmen: A case for the survival and evolution of the fittest. J. Sports Sci..

[22] Lupo C., Boccia G., Ungureanu A.N., Frati R., Marocco R., Brustio P.R. (2019). The Beginning of Senior Career in Team Sport Is Affected by Relative Age Effect. Front. Physiol..

[23] Rubia A., Bjørndal C., Sánchez-Molina J., Yagüe J., Calvo J., Marto-Izquierdo S. (2020). The relationship between the relative age effect and performance among athletes in World Handball Championships. PLoS ONE.

[24] Mon-López D., Tejero-González C.M., de la Rubia Riaza A., Calvo J.L. (2020). Pistol and Rifle Performance: Gender and Relative Age Effect Analysis. Int. J. Environ. Res. Public Health.

[25] Deaner R.O., Lowen A., Cobley S. (2013). Born at the wrong time: Selection bias in the NHL Draft. PLoS ONE.

[26] Fumarco L., Gibbs B.G., Jarvis J.A., Rossi G. (2017). The relative age effect reversal among the National Hockey League elite. PLoS ONE.

[27] Mankiw N.G. (2014). Principles of Economics.

[28] Ashworth J., Heyndels B. (2007). Selection bias and peer effects in team sports. The effect of age grouping on earnings of German soccer players. J. Sports Econ..

[29] Doyle J.R., Bottomley P.A. (2018). Relative age effect in elite soccer: More early-born players, but no better valued, and no paragon clubs or countries. PLoS ONE.

[30] Deloitte (2018). Football Money League.

[31] Tranfermarkt. https://www.transfermarkt.com/.

[32] Frick B. (2011). Performance, Salaries and Contract Length: Empirical Evidence from German Soccer. Int. J. Sport Financ..

[33] Herm S., Callsen-Bracker H.M., Kreis H. (2014). When the crowd evaluates soccer players’ market values: Accuracy and evaluation attributes of an online community. Sport Manag. Rev..

[34] Peeters T. (2018). Testing the Wisdom of Crowds in the field: Transfermarkt valuations and international soccer results. Int. J. Forecast..

[35] Doyle J.R., Bottomley P.A. (2019). The relative age effect in European elite soccer: A practical guide to Poisson regression modelling. PLoS ONE.

[36] Cohen A.S., Kim S.H., Wollack J.A. (1996). An investigation of the likelihood ratio test for detection of differential item functioning. App. Psychol. Meas..

[37] Gerdin G., Hedberg M., Hageskog C.A. (2018). Relative Age Effect in Swedish Male and Female Tennis Players Born in 1998–2001. Sports.

[38] Lagestad P., Steen I., Dalen T. (2018). Inevitable Relative Age Effects in Different Stages of the Selection Process among Male and Female Youth Soccer Players. Sports.

[39] Barnsley R.H., Thompson A.H. (1988). Birthdate and success in minor hockey: The key to the NHL. Can. J. Behav. Sci..

[40] Hill B., Sotiriadou P. (2016). Coach decision-making and the relative age effect on talent selection in football. Eur. Sport Manag. Q..

[41] Webdale K., Baker J., Schorer J., Wattie N. (2019). Solving sport’s ‘relative age’ problem: A systematic review of proposed solutions. Int. Rev. Sport Exerc. Psychol..

[42] Steingröver C., Wattie N., Baker J., Schorer J. (2016). Does relative age affect career length in North American professional sports?. Sports Med..

[43] Hancock D.J., Adler A.L., Côté J. (2013). A proposed theoretical model to explain relative age effects in sport. Eur. J. Sport. Sci..

[44] Frenger M., Follert F., Richau L., Emrich E. (2019). Follow me… on the relationship between social media activities and market values in the German Bundesliga. Work. Pap. Eur. Inst. Socioecon..

[45] Carreras M., Garcia J. (2018). TV Rights, Financial Inequality, and Competitive Balance in European Football: Evidence from the English Premier League and the Spanish LaLiga. Int. J. Sport Financ..

[46] Richau L., Follert F., Emrich M.F. (2019). Performance indicators in football: The importance of actual performance for the market value of football players. Sciamus Sport Manag. Jahrestag. Arb. Sportökonomie.

[47] Prockl F., Frick B. (2018). Information Precision in Online Communities: Player Valuations on www. Transfermarkt. De. Int. J. Sport Financ..

